# Recent advances in synthesizing and utilizing nitrogen-containing heterocycles

**DOI:** 10.3389/fchem.2023.1279418

**Published:** 2023-10-30

**Authors:** Hyun-Joon Ha

**Affiliations:** Department of Chemistry, Hankuk University of Foreign Studies, Yongin, Republic of Korea

**Keywords:** organocatalyst, pot-economy, tetrahydropyridine, (−)-quinine, environmentally benign

## Abstract

The use of organocatalysts and a pot economy has strengthened recent organic syntheses. Synthetic methodologies may be applicable in laboratory preparation or in the industrial production of valuable organic compounds. In most cases, synthetic challenges are overcome by highly efficient and environmentally benign organocatalysts in a pot-economical manner. This is exemplified by the recent synthesis of tetrahydropyridine-containing (−)-quinine.

In the last several decades, great progress has been made in organic synthesis. However, we are still quite far away from the ideal goal. In particular, synthetic reactions should be environmentally benign and should remove costly purification procedures derived from the step-by-step procedures used in a myriad of protecting and deprotecting protocols. In recent years, great success was achieved in overcoming these drawbacks by using several techniques, including fluorous catalysis (De Wolf et al., 1999; Mika and Horvath, 2018), solid-supported catalysis (Nafiu et al., 2023), biocatalysis (Pyser et al., 2021), and organocatalysis with green chemistry metrics (Martínez J. et al., 2022). Various trials with solvent-free and aqueous reactions (Ruiz-Lopez et al., 2020) or ionic liquids (Earle and Seddon, 2000) also succeeded in organic synthesis, with certain limitations. In addition, multicomponent reactions (Boukis et al., 2018; Cimarelli, 2019) were also applied based on pot-economical synthesis (Grondal C. et al., 2010). In recent years, great success has been achieved using a combination of organocatalysis and pot-economical synthesis. The one-pot synthesis of a target molecule in the same reaction vessel is widely considered to be an efficient approach in synthetic organic chemistry.

Although catalytic reactions have been known for a long time (List, 2010; Vogel et al., 2016), including various metallic compounds and a few organic molecules, no systematic investigation has been performed (Hajos and Parrish, 1974; Bernhard and Wolfgang, 1978). The concept and the term “organocatalyst” were established in earnest by the novel laurates B. List (List, 2007) and D. MacMillan (MacMillan, 2008) in 2007. They carried out various reactions, such as the addition of electron-deficient olefins like aldol and even Diels-Alder reactions (Chen et al., 2022). The advantages of organocatalysts include their lack of sensitivity to moisture and oxygen, making them easy to handle. In addition, most of them are readily available at low cost, with relatively low toxicity compared with metal catalysts (Albrecht et al., 2022). Diverse organocatalysts were developed as chiral catalysts to warrant the streamlined synthesis of optically active and/or pure products needed in many areas, including pharmaceutical industries (Hughes, 2018; Han, et al., 2021).

In terms of the great synthetic challenges of any organic compounds requiring many steps, all the necessary reactions should proceed in a highly efficient manner, including a pot-economy. A minimum reaction vessel required for the reaction to complete is an economical approach, such as in a pot economy. (Grondal C. et al., 2010). Reactions utilizing step-by-step protocols under different conditions require costly work-up and purification procedures. For example, the following flowchart shows three different reactions needed to get **D** as a product from three different reactions **I**, **II**, and **III**, yielding **B** and **C** as synthetic intermediate molecules. However, when we want to get **D** from the starting material **A** through several reactions, without isolating **B** and **C**, we are able to save laborious costly work-up and purification.

$$A\overset{\text{ReactionI}}{\to }B\overset{\text{ReactionII}}{\to }C\overset{\text{ReactionIII}}{\to }D$$

The best way to perform the different reactions in an efficient and easy way is to carry out all necessary reactions **I** to **III** in one pot under the same condition with the same reagents in the same solvent. For example, the synthesis of substituted 2,6-disubstituted piperidine from 3-alkynyl-2-(N-α-methylbenzylaziridine) was successfully achieved in the same way as shown in Scheme 1 by using a one-pot reaction under catalytic hydrogenation, without isolating any synthetic intermediate (Yadav et al., 2016). Under this condition, four different reactions, entailing the hydrogenation of alkyne, aziridine ring opening, reductive cyclization, and deprotection of the α-methylbenzyl group at the nitrogen ring occurred, without changing anything else throughout the whole reaction sequences.

**SCHEME 1:**
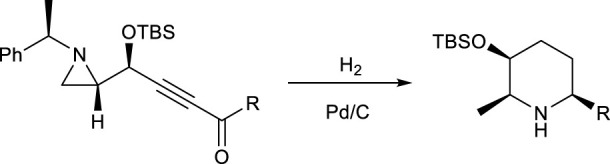
Synthesis of piperidine from multi-reactions of the starting material 3-alkynyl-2-(N-α-methylbenzyl)aziridine.

This is quite an exceptional case. In most cases, a preparative amount of product is accessible from reactions with different reagents under very specific conditions. Obtaining a decent amount of the final product in an efficient and handy protocol is possible with a one-pot synthesis. One-pot synthesis involves successive chemical reactions in just one reactor, from which sufficient purity is secured further in the sequence, without any purification. To carry out this synthesis properly, a decent reaction yield should be provided, with different reagents for the reaction to proceed to the specific position and functional group, even under different solvents. The reagents used for the previous reactions should not harm the subsequent reactions. For example, reactions **II** and **III** should be carried out by selecting proper reagents after killing the reactivity from the previous reactions to avoid harming the next reaction, i.e., reaction **II** from **I** and reaction **III** from both **I** and **II**. For this purpose, the synthetic intermediates in most cases are not stable for the next reaction, or they are in equilibrium for the next reaction. Selecting solvents is also a crucial factor for the specific reaction. In most cases, changing the solvents should be simple after one reaction, owing to the relatively low boiling points for them to be removed via simple evaporation.

For an efficient and environmentally benign synthesis, a pot-economical method is better for use in conjunction with organocatalysts. For example, the Michael reactions of nitroalkenes shown in Scheme 2 were investigated intensively, taking advantage of their superior reactivity toward the nucleophile and its practicability. Proper selection of organocatalysts derived from proline makes this transformation asymmetric, with high yields, and these are used in many cases. Though various organocatalysts were developed and utilized to make these reactions possible, diarylprolinol silyl ethers (**Cat**) are the most widely used as simple and very efficient catalysts. They carry out the reactions, including Michael reactions of nitroalkanes between **2** and **3**, in a highly efficient manner to yield products **4** with high optically purities (Y. Hayashi et al., 2005; Jensen et al., 2012; Reyes-Rodríguez et al., 2019) (Scheme 2). Once the key bond has been made, the others are followed by stereoselective reactions, with carbocyclic or heterocyclic ring transformations. The newly constructed bonds formed from organocatalysts are green, with specific examples indicated by the green-colored bonds in Scheme 2. In this manner, there are many reports showing that the organocatalytic reactions were very efficient ways of making the bonds needed in biologically important molecules, including pharmaceuticals and natural products such as acyclic and cyclic molecules. Some representative examples of organocatalyst proline derivatives, including diarylprolinol silyl ethers (**Cat**) are gabapentin (Gotoh et al., 2007), sacubitril (Hughes, 2018), prostaglandin PGF2α (Coulthard et al., 2012; Scheffler and Mahrwald, 2013), oseltamivir (Ishikawa et al., 2009; Ishikawa et al., 2010; Hayashi and Ogasawara, 2016), (+)-(α)-lycorane (Meng et al., 2014), (+)-microminutinin (Huang et al., 2017), and pyrrolysine (Han et al., 2013).

**SCHEME 2:**
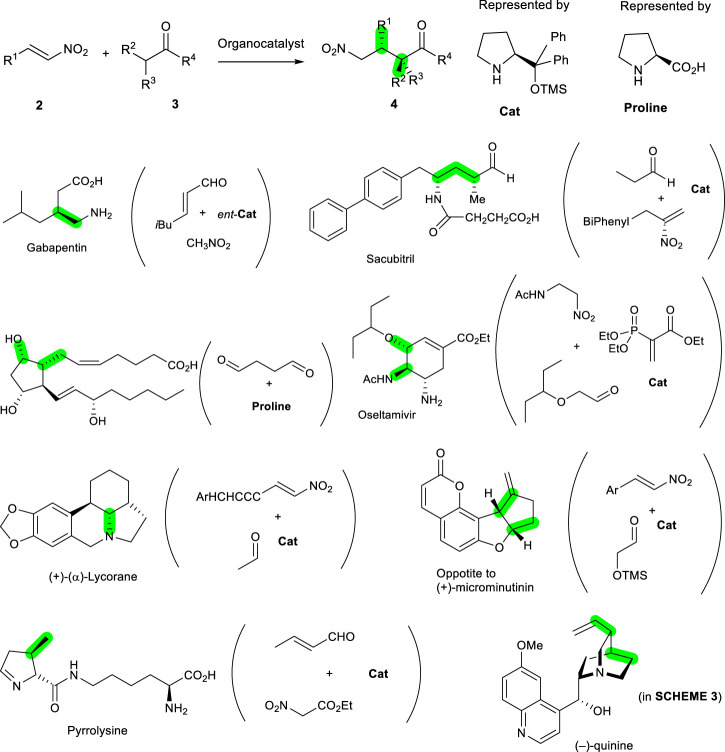
General synthetic scheme of bond formation and stereochemistry shown in green in adduct **4** based on organocatalytic nitro-Michael reactions from **2** and **3**. The catalyst used was proline or its diarylprolinol silyl ethers (**Cat**) derivatives. The specific cases of initial organocatalytic reactions with starting materials and catalysts are shown in parenthesis on the right side of the examples, with the construction of green-colored bonds.

Recently, one outstanding publication that is worth looking into in detail deals with the two important aspects, pot-economical and organocatalytic synthesis, and was carried out by Professor Y. Hayashi’s lab. They performed a very impressive synthesis of a (−)-quinine (**1**) natural product consisting of core azaheterocycle tetrahydropyridine (Terunuma and Hayashi, 2022). The synthesis, with a piperidine ring needed for the preparation of unnatural enantiomer, was previously published with an organocatalyst via (3 + 3) cyclization and Strecker-type cyanation (Shiomi, et al., 2019). However, a strategically different method was used to obtain azaheterocycle tetrahydropyridine as a core ring of the natural enantiomer (−)-quinine. The addition of aldehyde **5** to 2-phenylnitroalkene (**6**) for an aza-Henry reaction was initiated in the presence of 20 mol% of diphenyl prolinol trimethylsilyl ether as a catalyst (Scheme 3). Moreover, their synthesis was in conjunction with the pot-economical manner, leading to several reactions in the same vessel without any extra procedures or work-up (Hayashi, 2016). The imine needed to make a tetrahydropiperidine ring from the initial adduct between **5** and **6** would enhance the synthesis of **9** in the same vessel for a reaction completed in one pot.

**SCHEME 3:**
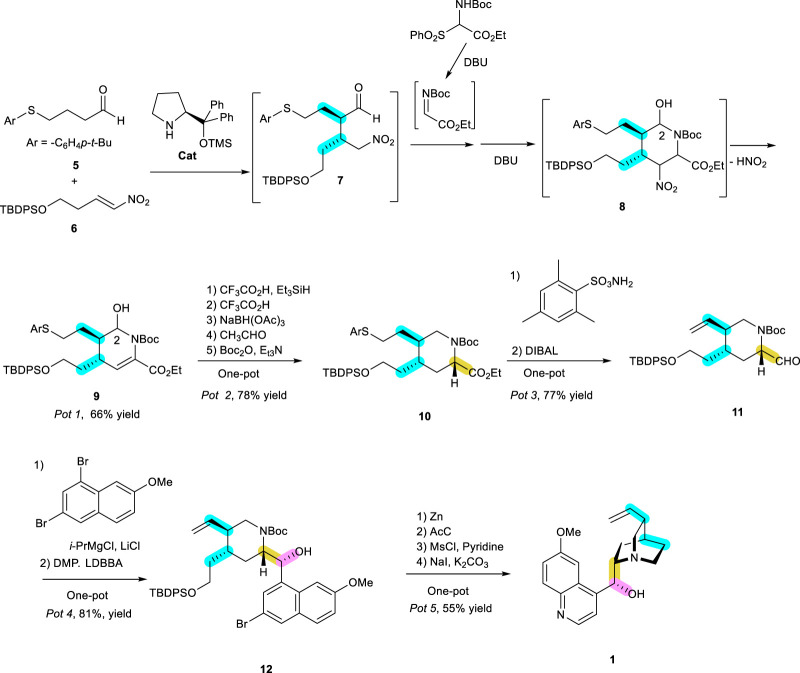
(−)-Quinine (**1**) synthesis reported by Professor Y. Hayashi’s lab (Terunuma and Hayashi, 2022) using an organocatalyst in a pot-economical manner as a key step in constructing the initial important bonds, highlighted in blue, followed by stereoselective reduction and aryl Grignard addition, as shown by the yellow and red bonds.

The initial acyclic adduct **7**, as a Niro-Michael product, was reacted with imine generated from the corresponding sulfonyl precursor DBU (1,8-diazabicyclo [5.4.0]undec-7-ene, 3.0 equiv) in a single pot manner. This synthetic intermediate was converted, with the elimination of HNO_2_ in the presence of DBU as a base, to yield the cyclic product **9** in high yield (66%) and high e. e. (98%), with 1:1 at C2 d.e. The hydroxy substituent at C2 is eventually eliminated. All the reactions starting from **5**, **6**, and the iminocarboxylate to **9** proceed sequentially in the one-pot protocol. To succeed in this reaction sequence, all synthetic intermediates were kinetically and thermodynamically favorable to afford the next product without any hindrance from the previous reagents, as shown in Scheme 3. The best way to perform this synthetic strategy is for all necessary reactions to succeed in a single operation without changing the reagents and solvents. However, this is very rare, with the assumption that the same reagents under the same solvent can carry out different reactions in a certain direction. Except for rare cases, almost all the reactions require different reagents under different solvents. Therefore, the reaction should be well designed to perform the steps in a pot-economical manner. As a diastereomeric mixture, the initial crude cyclic compound **9** from Pot 1 proceeded further for dehydroxylative reduction by CF_3_CO_2_H and Et_3_SiH. Then, the reduction of enamine to amine was followed by the reducing agent NaBH(OAc)_3_. All the excessive reductive reagents were destroyed by the reaction with additional acetaldehyde. This second one-pot reaction (Pot 2) afforded the azaheterocycle **10** as an almost single stereoisomer, with the correct stereochemistry. In Pot 3, it is relatively straightforward to obtain olefin **11** with the release of aryl thiol according to the Matsuo protocol (Matsuo et al., 2006) with 2,4,6-trimethylphenylsulfonylamide. The substituent carboxy ester was then reduced by DIBAL under low temperatures to give rise to aldehyde **11**. Then, 2-bromo-6-methoxyquinoline was added to this aldehyde, generating a diastereomeric mixture of hydroxy carbon without much stereoselectivity between *re*- or *si* face addition. However, this problem was overcome by oxidation of the alcohol adduct to ketone by DMP (Dess-Martin Periodinane), followed by the stereoselective reduction by LDBBA (lithium diisobutyl-*tert*-butoxyaluminum hydride), leading to the right isomer **12** needed for target **1** with more than 80% yield (Pot 4). The next steps in Pot 5 are the removal of bromine in the aryl ring and deprotecting the TBDPS and Boc groups from the free hydroxy group by treatment with Zn and AcCl. This primary hydroxy group was mesylated for the final cyclization with nitrogen to give quinuclidine under mild conditions using NaI and K_2_CO_3_. These four reactions are performed in a one-pot sequence with 77% yield. All these reactions are highly stereospecific, as seen in the synthesis of the expected (−)-quinine (**1**) in a pot economical manner. This report brings up two important and challenging synthetic methodologies in organic chemistry, i.e., the use of environmentally benign organocatalysts and pot economy.

This perspective focuses on the use of organocatalysts in a pot-economical manner for the recent synthesis of tetrahydropyridine-containing (−)-quinine. These synthetic methodologies may be applicable in laboratory preparation or in the industrial production of valuable organic compounds (Bernardi, et al., 2021). Most synthetic challenges are highly efficient and environmentally benign with organocatalysts in a pot-economical manner.

## Data Availability

The datasets presented in this article are not readily available because there are not any restrictions for readers to request. Requests to access the datasets should be directed to hjha@hufs.ac.kr.
